# A pilot study on community-based outpatient treatment for patients with chronic psychotic disorders in Somalia: Change in symptoms, functioning and co-morbid khat use

**DOI:** 10.1186/1752-4458-6-8

**Published:** 2012-07-02

**Authors:** Michael Odenwald, Birke Lingenfelder, Wolfgang Peschel, Farhan Adam Haibe, Abdirisak Mohamed Warsame, Ahmed Omer, Judith Stöckel, Anna Maedl, Thomas Elbert

**Affiliations:** 1 Department of Psychology, University of Konstanz, Fach D25, Constance, 78457, Germany; 2 Vivo, Zur Setze 4, Allensbach, 78476, Germany; 3 Institut of Psychology und Pedagogics, Clinical and Biological Psychology, University of Ulm, Albert-Einstein-Allee 47, Ulm, 89069, Germany; 4 Psychiatric Policlinic, St. Olav’s Hospital, Trontheim, Norway; 5 GAVO, Hargeisa, Somaliland

**Keywords:** Low and middle income countries, Severe mental disorders, Community-based mental health care, Schizophrenia, Khat

## Abstract

**Background:**

In Low and Middle Income Countries, mental health services are often poorly developed due to the lack of resources and trained personnel. In order to overcome these challenges, new ways of care have been suggested such as a focus on community-based services. In Somalia, the consumption of the natural stimulant khat is highly prevalent, aggravating mental illness. At the same time, mental health care is largely unavailable to the vast majority of the population. In a pilot project, we tested possibilities for effective measures in community-based out-patient mental health care.

**Methods:**

Thirty-five male patients with chronic psychotic disorders and their carers were involved in a 10-months follow-up study. All of them abused khat. Seventeen outpatients experiencing acute psychotic episodes were recruited from the community and received an intensive six week home-based treatment package. Additionally eighteen patients with chronic psychotic disorders in remission were recruited either following hospital discharge or from the community. In a second phase of the study, both groups received community-based relapse prevention that differed in the degree of the family’s responsibility for the treatment. The treatment package was comprised of psycho-education, low-dose neuroleptic treatment, monthly home visits and counseling. The Brief Psychiatric Rating Scale (BPRS) was applied three times. Additionally, we assessed functioning, khat use and other outcomes.

**Results:**

Of the 35 patients enrolled in the study, 33 participated in the 10-month follow-up. Outpatients improved significantly in the first six weeks of treatment and did not differ from remitted patients at the start of the second treatment phase. In the preventive treatment phase, we find heterogeneous outcomes that diverge between symptom and functioning domains. With the exception of depressive symptoms, symptoms in all patients tended to worsen. The outpatient group had higher BPRS positive and negative symptom scores compared to the remitted group. Levels of functioning in 20 out of 33 patients significantly improved, with small differences between groups. Most patients experienced improvements in basic functioning, such as communication, self-care *etc.* Khat use could only be reduced in the group of outpatients.

**Conclusions:**

Community-based out-patient mental health treatment for chronic psychotic disorders has demonstrated positive effects in Somalia and is both feasible and practical, despite facing formidable challenges, e.g. controlling khat intake.

## Introduction

Mental disorders are responsible for a large proportion of the global burden of diseases and premature deaths [1,2]. Especially in Low and Middle Income Countries (LMIC) where psychiatric services are poorly developed, there is a large treatment gap [3,4] and consequently a high burden of mental illnesses [5]. Chronic psychotic disorders constitute a group of disorders with especially high costs to individuals, families, communities and societies alike [6-8], especially in LMICs where the course and outcomes of the disorder remain debilitating [9,10]. In LMICs, mental health services are short staffed and often poorly developed [11]. High mortality rates among people with mental illnesses [9] are common and caregivers resort to inhumane practices such as forcible restraint [12].

Recently mental health experts have recommended new methods of mental health care in LMICs that are adapted to the local needs and resources, e.g. involvement of non-psychiatric staff, non-medical staff (e.g. nurses), and low dose neuroleptic treatment in the community [13,14]. In spite of initial attempts to implement community-based or non-specialist care for schizophrenia [15-17] the implementation keeps facing huge challenges [3,18-20].

After several decades of civil war as well as repeated drought and famine, Somalia is one of the world’s poorest countries and sadly holds the record on the Failed State Index [21]. The former British protectorate Somaliland declared independence in 1991, becoming the Republic of Somaliland and, although not internationally accepted, has developed with increasing stability in contrast to the rest of the country. We have observed high rates of chronic psychotic disorders among men in Somaliland and documented that the majority of these patients have a history of khat abuse [12]. Despite the elevated prevalence of psychotic and other mental disorders amongst the two to three million inhabitants [22], there are only a few providers of mental health services, each of which is understaffed and under-resourced [22,23]. The specific challenges of building a health care system in Somaliland have recently received some attention [24].

Khat leaves (khat; catha edulis) have been traditionally consumed in the countries around the Horn of Africa and are today the most commonly abused substance in Somalia. During the past 30 years, khat has had an enormous boom and its use dramatically increased in Somalia [25] while traditional social regulation mechanisms simultaneously eroded [26]. The principal agent cathinone is an amphetamine-like alkaloid [27] and was recently associated with compulsive use and addiction [28]. Excessive khat use can elicit psychotic symptoms and reactions [29,30]. Other mental disorders can increase the use of khat as means of self-medication with an increasing danger of psychotic developments [29,31]. In our previous cross-sectional studies of psychosis in Somalia, early onset of khat use and excessive use were retrospectively associated to the development of chronic psychosis [12]. Bimerew and colleagues found that khat use is related to the relapse of previously diagnosed schizophrenia [32].

In the present study, we piloted the implementation of different components of a community-based treatment study for patients with chronic psychotic disorders in Somalia: an acute treatment package and two variants of preventive long-term treatment. As all of the psychotic patients were khat users, additional consideration was given to co-morbid disorders. This co-morbidity reflects that in Somalia most men, particularly male psychotic patients, abuse khat [12]. The following research questions were asked: Is a home-based psychiatric treatment including neuroleptics feasible when implemented by paramedics lacking psychiatric expertise? Is a treatment implemented by paramedics ethically justifiable or even required, vis a vis a common practice of hog-tying humans? Is it possible for a community-based treatment program without hospital support to effectively reduce psychotic symptoms and improve functioning in patients who abuse khat? Can khat use be controlled among severely psychotic patients being treated in the community? Providing answers to these questions allows the development of innovative concepts for large-scale mental health service delivery, the identification of important factors that determine success and break ground for more extensive studies in the future.

We hypothesized that symptom severity can be significantly reduced within six weeks among acutely psychotic patients by receiving treatment exclusively within the community. Furthermore, we predicted that in the two models of continued psychiatric care for remitted patients, low symptom levels can be maintained, levels of functioning can be improved and khat use decreased over a period of ten months. Primary outcome measures included psychiatric symptom levels, every-day functioning and khat use; secondary outcome measures included Body Mass Index, medication side effects, medication compliance, restraint and the use of other mental health treatments during the project period.

## Methods

### Design

**Figure 1:**
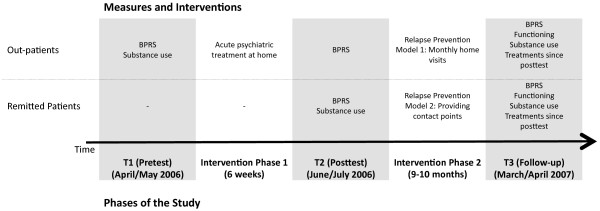
Design of the study.

In this intervention study, we followed up with two groups of male patients with chronic psychosis and their families: acute psychotic patients exclusively treated in their families within the community (from hereon named “outpatients”; N = 17) and patients with remitted psychosis (from hereon named “remitted patients”, N = 18) that were recruited either from the community (N = 7) or at discharge from inpatient treatment (N = 11). In the first phase of the study (acute treatment) only outpatients were involved, and we compared within symptom changes from pre to post acute treatment (six weeks). In the second phase of the treatment (relapse prevention), we compared the effect of continued psychiatric care in outpatients and remitted patients. In this phase of the study, outpatients received continued psychiatric care exclusively during home visits (outpatients) while for remitted patients, after an initial contact through service providers (e.g. inpatient services) home visits were designed to establish active responsibility of patients and their carers for medication intake (see Figure 1). We report data from three assessments: in April or May 2006 (T1), before inclusion into phase one of the study; six weeks later, in June or July 2006 (T2), after the first six weeks of acute outpatient treatment. At this point (T2), remitted patients entered the study (i.e. first assessment for remitted patients); and ten months later (T3), in March or April 2007(both groups; see Figure 1). Outcomes of later follow-up assessments will be reported elsewhere.

### Participants

In total, 41 male patients were approached and six patients could not be included in the project because other neurological or psychiatric disorders did not allow a neuroleptic treatment (N = 2) or patients and their carers refused to cooperate (N = 4). The outpatient group was recruited from the community and consisted of 17 patients with chronic psychotic disorders not receiving psychiatric treatment and experiencing an active illness episode. The second group comprised 18 patients who also suffered from chronic psychotic disorders that were in remission at that time: 11 patients were recruited at discharge from psychiatric in-patient treatment and 7 psychotic patients currently in remission who were recruited in the community and who currently did not receive any in-patient treatment. Patients who were recruited in the community were participants of an earlier cross-sectional study [12]. All patients fulfilled DSM-IV criteria A, B and C for Schizophrenia [33].

Socio-demographic characteristics are reported in Table 1. Both groups were comparable in all variables but Body Mass Index; patients recruited in the community were mal-nourished at the project start. In both groups we found very high levels of khat addiction (previous year) and an early age of khat use onset.

**Table 1:** Socio-demographic characteristics, BMI and information on substance use of enrolled patients

	**Outpatients (17)**	**Remitted patients**		
		**Total (18)**	**Discharged (11)**	**Community (7)**2
Age	36.5	36.4	38.2	33.7
	(9.8)	(8.0)	(8.0)	(7.7)
Years of education^2^	6.9	5.6	4.9	6.6
	(5.0)	(5.3)	(5.7)	(5.0)
Rating of economic situation index^3^	1.3	1.6	1.1	2.3
	(1.1)	(1.0)	(0.9)	(0.8)
Objective index of economic situation^4^	1.9	2.1	1.7	2.8
	(1.0)	(1.2)	(1.2)	(0.8)
Number of household members^5^	8.5	7.1	6.6	7.8
	(3.0)	(3.9)	(4.1)	(2.9)
Currently married^6^	12%	33%	46%	14%
	(2)	(6)	(5)	(1)
Refugee experience^7^	65%	69%	89%	43%
	(11)	(11)	(8)	(3)
Combat exposure^7^	29%	44%	56%	29%
	(5)	(7)	(5)	(2)
BMI at project start^8^	16.9	19.7	20.7	17.6
	(3.5)	(2.9)	(3.1)	(1.2)
Lifetime khat use^7^	94.1%	100%	100%	100%
	(16)	(17)	(10)	(7)
Age of onset of khat use^9^	15.8	17.3	17.1	17.6
	(4.6)	(4.1)	(4.2)	(4.2)
Khat addiction (last year)^10^	70.0%	93.3%	100%	80.0%
	(7)	(14)	(10)	(4)
Lifetime Alcohol use^4^	5.9%	18.8%	33.3%	0%
	(1)	(3)	(3)	(0)

Remitted patients comprise patients recruited at hospital discharge and in the community. The table reports means and standard deviations as well as percentages and N.

^2^ missing data: 17 outpatients, 17 remitted patients (10 discharged, 7 community).

^3^ missing data: 16 outpatients, 16 remitted patients (10 discharged, 6 community).

^4^ missing data: 17 outpatients, 16 remitted patients (10 discharged, 6 community).

^5^ missing data: 15 outpatients, 17 remitted patients (11 discharged, 6 community)

^6^ patients currently married as opposed to a composed category of being single, separated, divorced or widowed.

^7^ missing data: 17 outpatients, 16 remitted patients (9 discharged, 7 community).

^8^ missing data: 15 outpatients, 12 remitted patients (8 discharged, 4 community).

^9^ missing data: 15 outpatients, 17 remitted patients (10 discharged, 7 community).

^10^ missing data: 10 outpatients, 15 remitted patients (10 discharged, 5 community).

In Table 2, illness-related information is presented. On average, disruptive psychotic symptoms had emerged at the age of 24 years and the illness persisted for over 10 years. Outpatients had fewer episodes of in-patient treatment and their last hospital stay was longer ago than among remitted patients. Both groups had equally consulted different institutions for treatment, i.e. western-trained medical doctors, traditional healers (working with spirits or herbal medicines) or Islamic religious healers. Both groups had experienced inhumane ways of managing mental illness common for regions without access to medical treatment (e.g. being imprisoned, chained, i.e. hog-tied, or locked up; for details see Table 2).

**Table 2:** Illness-related information

	**Outpatients (17)**	**Remitted patients**		
		**Total (18)**	**Discharged (11)**	**Community (7)**
Age at psychosis onset	22.8 (7.3)	25.4 (6.2)	27.4 (6.3)	22.3 (4.9)
Duration of illness	13.6 (5.2)	11.0 (6.4)	10.7 (6.2)	11.4 (7.1)
Times treated in Mental Hospital	0.9 (0.9)	2.3 (2.7)	2.7 (3.3)	1.6 (1.1)
Last discharge from Mental Hospital: years ago^2^	3.2 (2.6)	1.0 (2.8)	0 (0)	3.2 (4.6)
Times consulted a medical doctor^3^	0.9 (1.1)	0.7 (0.8)	0.3 (0.5)	1.3 (1.0)
Times consulted a traditional healer^4^	1.7 (3.2)	1.9 (2.0)	2.3 (2.2)	1.4 (1.8)
Times consulted a sheikh^5^	1.6 (2.3)	1.2 (1.3)	1.7 (1.5)	0.6 (0.5)
Times admitted to prison due to mental problems^6^	1.8 (3.5)	0.9 (2.0)	0.9 (1.9)	1.0 (2.5)
Months of life locked up due to mental problems^7^	4.0 (14.6)	1.0 (3.6)	1.7 (4.6)	0.1 (0.0)
Months of life chained (hog-tied) due to mental problems^8^	57.8 (96.7)	15.9 (27.9)	4.1 (5.5)	32.9 (38.3)

Remitted patients comprise patients recruited at hospital discharge and in the community. The table reports means and standard deviations as well as percentages and N.

^2^only includes patients with a psychiatric inpatient history: 9 outpatients, 16 remitted patients (11 discharged, 5 remitted).

^3^missing data: 17 outpatients, 17 remitted patients (10 discharged, 7 community).

^4^missing data: 17 outpatients, 16 remitted patients (9 discharged, 7 community).

^5^missing data: 17 outpatients, 16 remitted patients (9 discharged, 7 community).

^6^missing data: 17 outpatients, 16 remitted patients (10 discharged, 6 community).

^7^missing data: 17 outpatients, 15 remitted patients (9 discharged, 6 community).

^8^missing data: 16 outpatients, 17 remitted patients (10 discharged, 7 community).

### Diagnostic process, measurement and instruments

In order to ensure the existence of a chronic psychotic disorder, items from the WHO’s Composite International Diagnostic Interview, psychosis part [34] were used at study entry. Additionally illness-related information was gathered in interviews with carers. Before study entry, khat addiction was assessed using the MINI International Neuropsychiatric Interview [35]. Several studies have documented that khat users can develop khat-related dependence syndrome [36,37].

At pre-, post- and follow-up test the Expanded Brief Psychiatric Rating Scale [38,39] was conducted to quantify the severity of related symptom clusters. This interview is a frequently used instrument with good psychometric properties. Besides the total score (all 24 items), we analyzed subscales based on the extensive study by Velligan and colleagues [40], i.e. Psychosis/Positive Symptoms (items: Unusual Thought Content, Hallucinations, Conceptual Disorganization, Bizarre Behavior, Cronbach’s Alpha .67), Negative Symptoms (Blunted Affect, Psychomotor Retardation, Emotional Withdrawal, Alpha .79), Depression/Anxiety (Depression, Anxiety, Guilt, Suicidality; Alpha .78), Activation (Excitement, Tension, Mannerisms and Posturing, Motor Hyperactivity; Alpha .68). In order to improve the legibility, we subtracted 1 from the original BPRS values. Structured clinical interviews were conducted by a trained senior psychiatrist (WP) and a trained senior psychologist (MO).

Before inclusion into the project we assessed whether the patient had ever used khat in his life. In every interview, we assessed khat use in the month prior to the interview as a categorical variable. Additionally, we quantified khat use in the week prior to the interview by days with khat use and by the amount of consumed traded units (bundles). We also used a simple amphetamine quick test (immunochromatographic assay) to determine the existence of khat alkaloids in the urine (Oekonomed, Darmstadt, Germany). The test has been evaluated for khat alkaloids in a previous study [41]. Despite the varying potency of khat samples and the limited comparability of bundles, there is currently no other feasible method to retrospectively quantify khat use [31]. This kind of test is considerably cheap (approximately half a dollar per test stripe) which is a prerequisite for the application in a Low Income Country.

At T3 we asked carers to generally rate whether the patients had improved in different domains of everyday functioning since inclusion into the project. In order to validate these yes-no questions, we additionally asked them to mention and describe activities of daily life in which they felt the patient had improved and behaves the way a healthy individual would (for example, talking to others, personal hygiene). For each activity we asked for the number of days in the previous week the patient demonstrated this improved behavior. Furthermore, we asked them on how many days per week the patient had behaved like this one year ago before the start of the project. We report the number of activities that had improved and the average number of days that had improved, i.e. the difference between last week and before project start. In order to estimate the validity of this assessment of functioning, we used data recorded from a different study. Carers’ functioning ratings were summed up by adding up all days last week functioning “as a healthy individual” over the different domains reported here; this sum scale achieved a moderate reliability (Cronbach’s Alpha .677). The sum scale correlated negatively with the total number of days hog-tied in the month before (−.489, p = .006), evincing that we have validly assessed levels of functioning.

Additionally, we assessed medication compliance (days without medication in the previous month), the way of a family’s management of patients in the last month (chaining, i.e. hog-tying, locking up) and treatment seeking behavior (consultation of traditional healers, religious healers and western trained medical doctors). Before inclusion into the project we quantified lifetime experience of being restrained due to mental problems (months chained, locked up or kept in prison). In Somalia, persons with mental illness and aggressive behaviors are frequently brought to prison by their families as an attempt to prevent offenses. As a measure for patients’ physical condition we used the BMI.

The economic situation of a household was rated using a five-point Likert scale (very poor, poor, moderate, good, very good). Additionally the wealth was assessed with a method reported by Odenwald et al. [12]. The sum of four significant household assets (electricity, water tap, TV set, car) and the house type (hut = 1, corregated iron = 2, brick house = 3, closed compound = 4) was assessed. The average served as indicator for the economic situation. This rating achieved a good realibility (Cronbach's Alpha = 0.78) and a high correlation with income (0.73, p < 0.001; [12]).

### Psychosocial care and medication

All patients included in the project received a free treatment package including neuroleptic medication. They were first offered low-dose neuroleptic treatment with Chlorpromazine (CPZ) as recommended by the WHO [42]. This substance was chosen based on an assessment of prescriptions in the Hargeisa Group Hospital psychiatric ward and of a local market survey before project start: CPZ proved to be the standard neuroleptic treatment in Somaliland and the only neuroleptic drug with an uninterrupted supply on the local market; its price proved to be stable on a low level, which is important for sustainability, as it might be necessary to continue the treatment beyond the duration of the project. Treatment was changed to low-dose Haloperidol in case CPZ was not tolerated.

Prescriptions were made on the basis of the diagnostic interviews and when available on medical records and information from the Hargeisa Group Hospital staff. For the discharged patients, after a thorough examination by a senior psychiatrist (WP), the hospital treatment regimen was adjusted when necessary and continued. Patients without any active illness process who were currently off any medication were offered preventive long-term treatment with very low doses. The average CPZ dose prescribed was with an average of 115 mg low (Table 3) in order to minimize side effects in the absence of an intensive psychiatric monitoring during the project phase. Adjunct treatment with Promethazine (9) or Biperidene (8) was prescribed when necessary. After follow-up, in two patients the neuroleptic treatment had to be changed to Haloperidol (2 mg/day) due to side effects (1) and missing effectiveness (1). All drugs were provided by the project and made available free of charge to the patients. A local general practitioner was contracted to be available during the project period in the case of arising questions concerning medication, side effects and co-morbid medical problems. Regular online supervision by senior researchers with local staff members assured that questions arising from home visits could be discussed and resolved.

**Table 3:** Treatment delivery of the project

	**Outpatients (17)**	**Remitted patients**			**p**^**1**^
		**Total (18)**	**Discharged (11)**	**Community (7)**	
N^o^ of home visits in 1^st^ 6 weeks after inclusion into the project	4.5 (2.1)	2.3 (1.1)	2.6 (1.0)	1.9 (1.2)	<.001
Prescricption of Chlorpromazine (mg)	122.1 (49.9)	105.6 (80.2)	145.5 (68.8)	42.9 (53.5)	.473
Prescription of additional Biperidene	41% (7)	6% (1)	9% (1)	0% (0)	.018
Prescription of additional Promethazine	35% (6)	17% (3)	27% (3)	0 (0)	.264

The table reports means and standard deviations as well as percentages and N.

^1^comparison of outpatients and remitted patients.

### Treatment delivery

At the time of inclusion into the project, both groups received repeated home visits by international research staff for confirmation of diagnosis, prescriptions of psychopharmacological drugs and instruction on the project. The acute treatment phase of the project involved only the group of outpatients. In the acute treatment phase, team members carried out a total of 77 home visits for diagnostic interviews, prescription and adjustment of medication by an experienced psychiatrist (see Table 3). In case additional medical problems emerged, patients and their carers were offered assistance to find adequate medical treatment. In this first treatment phase, families and patients received intensive information on the negative effects of khat use on psychotic disorders; special emphasize was put on motivation to stop using khat. T2 was the posttest for outpatients and measured the effect of the acute treatment in this group. T2 was the first assessment of remitted patients, who entered the study in a low-symptom phase of their illness, and marked the beginning of the preventive phase of treatment. Remitted and outpatients were enrolled in the second, preventive treatment phase of the study. At the time of inclusion into the study, remitted patients received a total of 41 home visits for diagnostic interviews and continuation of prescription.

In the second phase of the study, outpatients and remitted patients received a total of 196 home visits by trained staff of our local partner NGO GAVO. Home visits were the central element of the treatment packages delivered to both groups. The home visits in both groups included the monitoring of symptoms and side effects, repeated psycho-education, motivation to adhere to the prescribed treatment, the discussion of current problems, active listening to the carers’ and patients’ problems and emotional support. Local research staff was also instructed to motivate patients and carers of both groups to stop using khat.

Additionally, the two treatment packages differed in important aspects. Outpatients and their carers had a much lower responsibility for their medication treatment as compared to remitted patients and their carers. Outpatients received the prescribed drugs during home visits by the staff of our partner NGO. This implied that a very close monitoring of medication intake took place, i.e. tablets were counted precisely and the prescription regimen was explained and discussed in detail during every home visit with both carers and patients. Non-compliance was noticed early and team members tried to convince patients and carers or supported carers to improve the patient’s compliance. In case of emerging side effects team members assisted by consulting with the supervising psychiatrist (online supervision) and were able to adjust doses or start with adjunct treatment.

On a monthly basis, remitted patients and their carers were responsible for obtaining their medication from a location several kilometers from home. The group of discharged patients received their medication on a monthly basis from the hospital dispensary where had been treated before and where they had to wait in long lines during opening hours; the remitted community patients were asked to collect their medication from the office staff of our local partner NGO (not from the social workers who did the home visits). The reason for this type of medication delivery was the observation that it is beneficial for patients and their families to maintain contacts to the institution that provided in-patient care. As a consequence of this different form of medication delivery, after briefly asking for whether problems with medication existed, home visits among remitted patients were mainly focused on other topics and were much shorter. In the case of most remitted patients our partner NGO’s staff was not aware of the exact extent of problems with medication compliance.

In our observation, treatment delivery different also in the following aspects: In the outpatient group home visits were much longer and more intense. Because only outpatients participated in the acute treatment phase, the relationships that developed between project staff, patients and carers were more intensive.

### Ethical approval

Prior to inclusion into the project, patients and their carers were fully informed about the project and were asked to give their written informed consent to participate in the project. We especially highlighted that benefits related to the project were exclusively psychiatric treatment to the patient and psycho-social assistance to the patient and the carer and NOT economic support.

All procedures had been approved in 2006 by the University of Konstanz Institutional Review Board. Additionally, the project was approved by the Somaliland Ministry of Health and Labour and the National Health Research Ethical Committee.

### Statistical analysis

Changes of single variables over time were tested using paired t-tests. For testing changes of psychotic symptoms over the various assessments we performed repeated measures ANOVA for the group of outpatients and remitted patients separately. We report Greenhouse-Geisser corrected values whenever the sphericity assumption was violated.

We compared the two patient groups using Students *t*-test (or Mann–Whitney test in case preconditions were not fulfilled) and chi^2^ test (or Fisher’s exact test if preconditions were not fulfilled). If not otherwise reported, a One-Way-ANOVA (or Kruskal-Wallis test if preconditions were not fulfilled) comparing outpatients with the two subgroups of remitted patients (discharged, community patients) and Scheffe’s post-hoc tests (or Mann–Whitney if preconditions were not fulfilled) did not reveal significant differences.

## Results

At follow-up, 33 of our 35 patients could be traced and re-interviewed (17 outpatients and 16 control patients).

### Side effects

According to the ratings of patients and their carers at follow-up, most patients (87%) experienced side effects of the psychiatric medication; the number of patients reporting any side effects did not differ between the groups (p = .613). These side effects were usually tolerable and the contracted medical doctor did not need to be consulted in any case of side effects. Severity did not differ between outpatients and remitted patients (see Additional file 1: Table S1; p = .794). In total, 40% (12) of the patients experienced side effects that were rated severe, this was mostly sedation, increased saliva production and dyskinesia; the prevalence of severe side effects did not differ between groups (.547).

### Medication compliance, other treatments and restraining

According to carers, 63% of patients always took their medication voluntarily during the project (12/17 outpatients vs 7/13 remitted patients, p = .454). In the last month before follow-up, 61% of patients were compliant with medication (13/17 outpatients vs 7/16 remitted patients, p = .055). We found a strong trend that outpatients were compliant to prescribed medication on more days in the month before follow-up interview than remitted patients. The former did not take medication for on average 6.5 days (SD 10.9) while the latter for 14.9 days (SD 13.8; p = 0.058).

At the time of inclusion into the project, ten patients were chained in their homes, six of them in the group of out-patients, four among remitted patients (p = .708). We found that chaining of remitted patients was a common practice especially in situations in which families did not fully trust in the patients’ symptom stability and khat abstinence. At the time of the follow-up interviews, eight patients were chained, five of them among the outpatients and three among remitted patients (p = .688). Compared to project start, we found at follow-up one patient had been newly chained and three were released. During the follow-up period, six of the ten patients chained at project start and one other patient who had been newly restrained had been repeatedly released and re-restrained; this circle of chaining, release and re-restraining happened up to 5 times, on average one time per participant; outpatients and remitted patients were equally often ever restrained during the project.

On average, study participants remained restrained at their homes for 60.1 days (SD 113.6) during the second project phase; outpatients and remitted patients did not differ in this respect (116.9 days SD = 28.4 vs 113.9 days, SD = 29.4; p = .939; patients not having been chained excluded).

Four remitted patients and none of the outpatients were admitted for in-patient treatment to the psychiatric ward of the Hargeisa Group Hospital during the follow-up period (p = .044). Average length of stay was 35.6 days (SD 73.7, range 60–240 days); all of them were still hospitalized at the time of follow-up interviews.

During the follow-up period ten patients contacted a medical doctor because of psychiatric problems, three among the outpatients and seven among the remitted patients (p = .259). On average, patients spent 1.1 US$ (SD 2.8) for these medical treatments, ranging from 0 to 10 US$ per consultation; there were no differences between outpatients and remitted patients (0.88 US$, SD 2.6 vs 1.3 US$, SD 3.1; p = .647).

During follow-up five patients contacted traditional healers or sheikhs, two in the remitted group, three in the outpatient group (p = 1.000) and spent between 40 and 100 US$ per consultation. On average, patients spent 10.9 US$ for their treatments (SD 28.0); outpatients spent on average 14.1 US$ (SD 33.8), remitted patients 7.5 US$ (SD 20.8; p = .506). Between those patients who had contacted a sheikh or healer and those who did not, no statistical significant differences regarding days without medication in the month before the follow-up interview emerged (16.6 days, SD 15.4 vs 9.3 days, SD 12.3; p = .448).

Nineteen of the study participants did not search for assistance outside to what was offered from the project, while 15 did (four participants were receiving additional assistance from two types of services); outpatients and remitted patients did not differ regarding any contact with service providers outside the project (p = .119) and number of different service providers approached (0.35, SD 0.61 vs 0.75, SD 0.77; p = .110).

### BMI

At the time of inclusion into the study, 21 patients had a BMI below 20 and 9 below 16. At that point in time, the BMI of outpatients was significantly lower than that of remitted patients (p = .039; see Table 1). This difference was due to lower BMI in all patients recruited in the community. At follow-up, no difference could be detected any more (outpatients: M = 19.3, SD = 3.6; remitted patients: M = 21.2, SD = 2.7; p = .123). In both groups, significant increases of the BMI were detected from pre-enrollment to follow-up assessment (p = .001, p = .008). At follow-up, 14 patients remained with a BMI below 20, but only 2 below 16.

### Changes in symptom severity

Outpatients initially had a moderate to severe psychotic symptom profile (Table 4). Before the inclusion into the acute treatment phase we found a moderate correlation of khat use as measured by the urine test to BPRS subscale Positive Symptoms (.430, p = .085, n = 17). Correlations to other BPRS scales were much smaller (r < = .296, p > = .266).

**Table 4:** BPRS symptom ratings at pre, post and follow-up assessments

		**Outpatients (17)**	**Remitted patients (18)**	**Between-group comparison**
				**p**
BPRS Total	Pre	1.43 (0.59)	-	-
	Post	0.27 (0.47)	0.43 (0.38)	.273
	Follow-up^2^	1.47 (0.69)	0.90 (0.67)	.021
Repeated measures ANOVA	p	<.001^3^	<.001^4^	-
	Contrasts	Pre-Post: <.001	-	-
		Pre-FU: .816		
		Post-FU: <.001		
BPRS Positive	Pre	1.70 (1.05)	-	-
	Post	0.36 (0.63)	0.32 (0.51)	0.823
	Follow-up^2^	1.81 (1.47)	0.89 (1.15)	.054
Repeated measures ANOVA	p	<.001^5^	.027^6^	-
	Contrasts	Pre-Post: <.001	-	-
		Pre-FU: .791		
		Post-FU: .001		
BPRS Negative	Pre^7^	1.56 (0.83)	-	-
	Post	0.32 (0.68)	0.37 (0.46)	.812
	Follow-up^2^	2.89 (1.71)	1.42 (1.13)	.024
Repeated measures ANOVA	p	<.001^8^	<.001^9^	-
	Contrasts	Pre-Post: <.001	-	-
		Pre-FU: .022		
		Post-FU: <.001		
BPRS Depression	Pre^7^	2.06 (1.43)	-	-
	Post	0.34 (0.70)	0.64 (0.47)	.144
	Follow-up^2^	0.50 (0.79)	0.65 (0.60)	.562
Repeated measures ANOVA	p	<.001^10^	.974^11^	-
	Contrasts	Pre-Post: <.001	-	-
		Pre-FU: <.001		
		Post-FU: .551		
BPRS Activation	Pre^12^	1.44 (0.72)	-	-
	Post^13^	0.49 (0.58)	0.38 (0.52)	.684
	Follow-up^2^	0.90 (1.12)	0.70 (0.94)	.595
Repeated measures ANOVA	p	.007^14^	.444^15^	-
	Contrasts	Pre-Post: <.001		-
		Pre-FU: .051		
		Post-FU: .120		

The table reports means and standard deviations of different BPRS scales and outcomes of within-group as well as between-group comparisons.

^1^comparison of outpatients and remitted patients.

^2^missing data: 17 outpatients, 16 remitted patients (10 discharged, 6 community).

^3^repeated measures ANOVA: df = 2, F = 32.252.

^4^repeated measures ANOVA: df = 1, F = 42.780.

^5^repeated measures ANOVA: df = 2, F = 10.308.

^6^repeated measures ANOVA: df = 1, F = 6.031.

^7^missing data: 16 outpatients.

^8^repeated measures ANOVA: df = 1.260, F = 18.428.

^9^repeated measures ANOVA: df = 1, F = 20.950.

^10^repeated measures ANOVA: df = 2, F = 20.757.

^11^repeated measures ANOVA: df = 1, F = 0.001.

^12^missing data: 10 outpatients.

^13^missing data: 17 outpatients, 6 remitted patients (4 discharged, 2 community).

^14^repeated measures ANOVA: df = 2, F = 6.629.

^15^Repeated measures ANOVA: df = 1, F = 0.771.

**Figure 2:**
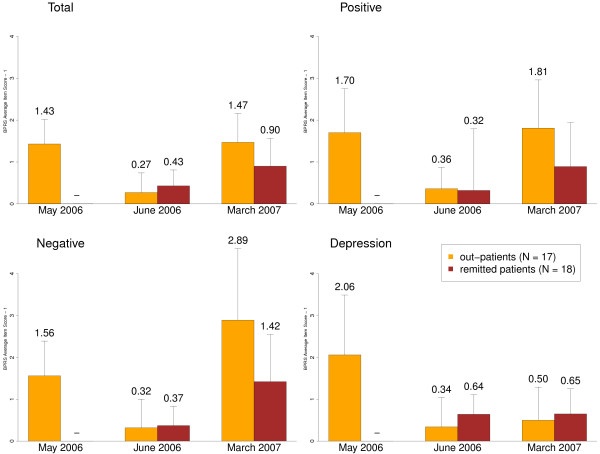
**Development of psychotic symptom severity during the project.** We report BPRS scales for the different assessment times. Bars represent means and standard deviations. In order to improve the legibility we subtracted 1 from original BPRS values.

After the first six weeks of treatment in their families, symptom levels of outpatients had significantly improved (p < .001 for all BPRS scales; see Table 4), reaching the level of the remitted patients, i.e. no significant differences in all BPRS scales between the two groups (see Table 4; Figure 2). Remitted patients who entered at this stage into the study showed negative correlations between the khat urine test and BPRS scales (Total -.460, p = .073, Positive -.358, p = .174, Negative -.573, p = .020, Depression -.427, p = .099; N = 16).

In the follow-up assessment, symptom severity had increased in both groups as indicated by significant higher BPRS scales. Comparing the different BPRS scales using repeated measures ANOVA, the deterioration of symptoms in both groups at follow-up compared to T2 emerged in the Total scale, the Positive Symptoms and Negative Symptoms subscales but not in the Depression/Anxiety subscale where improvements could be maintained (see Table 4). This deterioration was more pronounced in the outpatient group as indicated by significantly or nearly significant greater BPRS Total, Positive and Negative Symptom scores compared to remitted patients (see Table 4). At follow-up, we found negative correlations between the khat urine test and BPRS scales without differences between groups (Total -.419, p = .019, Positive -.467, p = .008, Negative -.029, p = .878, Depression, -.266, p = .156, Activation, -.197, p = .288; N = 31).

### Improvement in every-day functioning

Our second primary outcome measure for treatment success was the improvement in every-day functioning (Table 5). In the opinion of 65% (N = 20/31) of the carers, the project led to an improvement in the every-day functioning of the patients. We also asked for specific domains of improvement (see Table 5). Most notably, 80% (N = 24/30) of carers affirmed a reduction in aggressive behaviors; carers rated this improvement more frequent among outpatients than among remitted patients (p = .019). Other domains of functioning with substantial improvement were social conduct in general (67%), insight into illness (55%) and reduction of khat use (57%).

**Table 5:** Carers’ ratings of functioning improvement

	**All patients (35)**	**Outpatients (17)**	**Remitted patients (18)**	**p**^**1**^
General improvement^1^	64.5% (20)	70.6% (12)	57.1% (8)	.477
Social behavior improvement^2^	66.7% (22)	76.5% (13)	56.3% (9)	.218
Insight into illness improved^2^	54.5% (18)	47.1% (8)	62.5% (10)	.373
Less aggression^3^	80.0% (24)	100% (14)	62.5% (10)	.019
Reduced khat chewing^4^	57.1% (16)	66.7% (8)	50.0% (8)	.378
Tolerated khat better^5^	51.9% (14)	66.7% (8)	40.0% (6)	.168
Improved insight into dangers of khat^3^	26.7% (8)	21.4% (3)	31.3% (5)	.689
Improved productive life^6^	45.5% (15)	47.1% (8)	43.8% (7)	.849
Other improvement^7^	9.4% (3)	18.8% (3)	0 (0)	.226
Caretaker states any improved every-day activity^6^	60.6% (20)	76.5% (13)	43.8% (7)	.055
Number of improved every-day activities^6^	1.48 (1.46)	1.82 (1.42)	1.13 (1.45)	.173
Average number of days with improved activities^6^	3.20 (3.07)	3.62 (2.89)	2.75 (3.28)	.423

We report percentages and n (in brackets) or means and standard deviations (in brackets).

^1^missing data: 17 outpatients 14 remitted patients.

^2^missing data: 17 outpatients 16 remitted patients.

^3^missing data: 14 outpatients 16 remitted patients.

^4^missing data: 12 outpatients, 16 remitted patients.

^5^missing data: 12 outpatients 15 remitted patients.

^6^missing data: 17 outpatients, 16 remitted patients.

^7^missing data: 16 outpatients 16 remitted patients.

In order to validate these general yes-no questions, we asked carers to state every-day activities, which had improved since the time before the project. Twenty carers (61%; i.e. 20/33) were able to name such concrete improvements. The notion that patients improved in general was highly associated with mentioning any such improved activity (Phi = .518, p = .004). On average, carers mentioned 1.5 (SD = 1.5) such activities that were on average each related to an improvement of functioning of 3.3 days per week (SD = 3.1). There were no differences between groups of patients (Table 5).

In four cases (12.5%; 2/16 outpatients vs 2/16 remitted patients, p = 1.000) the improvements reported by carers were substantial, whereby former patients had become fully functional in their social environment. Three of them (1 outpatient and two remitted patients) could (re)start income-generating activities for 6 or 7 days per week; two of them became the major income providers of their families; another outpatient was unemployed despite restored functioning. In his own opinion the reason for this was his low educational level.

For the other patients, reported improvements were more basic, however, still important: carers reported that eleven patients (34.4%; 7/16 outpatients, 4/16 remitted patients, p = .264) had started to help with household or family duties, such as fetching water, doing shopping, helping with animals or farming *etc.*; the average number of days they had improved their respective behavior at follow-up was 5.4 days per week (SD = 1.9).

Nine patients (28.1%) normalized their basic communication and socializing behavior with an average improvement of 5.3 (2.5) days per week; in some cases they had not spoken at all for long periods and under medication started to interact again with their carers. Eight of the nine patients were outpatients (p = .015).

Seven others (21.9%; 4/16 outpatients vs. 3/16 remitted patients, p = 1.000) had substantially improved their personal hygiene and started to care for themselves, e.g. taking a shower or using a toilet; on average number of days with improved functioning in this domain was 6.1 (SD = 1.5).

The sum of reported improvement days were equally distributed between outpatients and remitted patients (p = .264)

### Khat use

**Figure 3:**
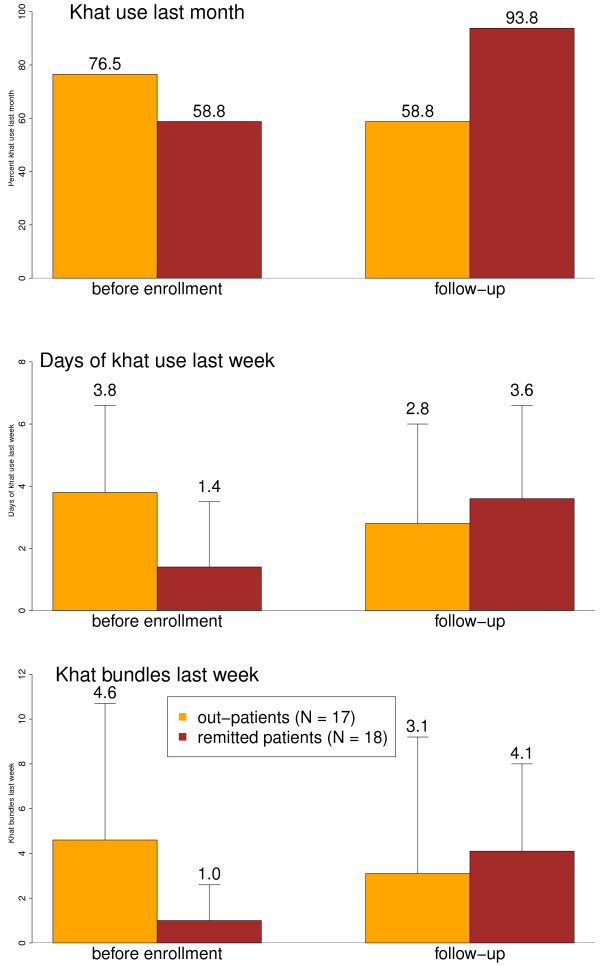
**Khat use indicators during the project.** Error bars represent percentages or means and standard deviations before enrollment and at follow-up.

Khat use was our third main outcome variable. In the month before inclusion into the project, 76.5% of outpatients and 58,8% of remitted patients had chewed khat (Figure 3; Additional file 1 Table S2; p = .271). In the week previous to the inclusion into the project, outpatients were using khat on more days than remitted patients (p = .013); the consumed amount in this week did not differ between the groups (p = .128), however, more excessive users could be found among the previous. In March/April 2007 (T3), almost all remitted patients had resumed khat chewing, more than outpatients (10/17 vs 15/16; p = .039). While we found a reduction of khat use as categorical variable in the course of the project among outpatients (p = .015), no significant change could be observed among remitted patients (p = .333). At T3, days of khat use and amount of bundles consumed in the last week did not differ between groups (p = .460, p = .594). In repeated measures ANOVAs the time*group interaction was nearly significant for the dependent variable khat bundles (p = .079) and significant for days with khat use in the last week (p = .014). In paired t-tests, the days of khat use as well as the consumed bundles in the previous week increased among remitted patients (p = .030, p = .016) but did not change among outpatients (p = .192, p = .543).

## Discussion

We studied and compared the feasibility and effects of family-based acute treatment and two models of community-based relapse-preventive outpatient care for patients with chronic psychotic disorders who abuse khat in Somalia. Patients with chronic psychotic disorders and their carers accepted the project and appreciated the assistance, as limited it was. In most cases, families worked reliably and closely with the project. Initial exaggerated hopes and expectations towards economic assistance could be dealt with in the course of the project. Our outcome variables showed a disassociation between psychotic symptoms versus levels of functioning and depression - the latter improved while the former did not. We also found that khat use can be reduced even among users with an addictive use pattern. In spite of the methodological shortcomings, our findings demonstrate that community-based psychiatric care in a country with almost no psychiatric resources can improve functioning and depressive symptoms in patients with long-term disorders living in inhumane conditions and is therefore ethically justified. Additionally, they also demonstrate that more stringent research is needed to scientifically prove evidence and that concepts of community-based mental health care need to be further developed.

The low dose treatment with Chlorpromazine seemed to produce a measurable improvement of the symptom levels, especially in the acute treatment phase, and clearly positive effects on every-day functioning and it proved to be relatively safe in respect to medical complications and side effects.

We found that acute treatment of productive psychotic patients in their homes where many of them were chained is effective, i.e. acute symptom levels can be reduced to a level comparable to patients in remission. Similar results are known from Soteria [43] treatment approaches using low-dose medication and home-based treatments in western countries [44].

Concerning relapse prevention in the community, our results go along the lines of other treatment studies showing that relapse cannot be fully prevented but its risk can be reduced even by low-dose neuroleptic treatment [45] and that first-generation anti-psychotics can be equally effective than newer drugs [46]. Of course, due to the lack of an untreated control group (i.e. treatment as usual), the effect of our interventions cannot be fully evaluated. Furthermore, it is a challenge to define relapse and remission in the setting in which we operated, because we lacked a continuous presence of resources and highly trained staff who would have been able to rate symptoms with clinical instruments. For example, in the dearth of in-patient services, the simple readmission criteria do not appropriately apply. We reduced this question to the measurement of symptom severity on a group basis at pre-, post and follow-up assessments. We found that both positive and negative symptoms improved during acute treatment and deteriorated again in the course of the follow-up period. Changes of positive symptoms from post to follow-up probably were influenced by khat use and medication compliance. Changes of negative symptoms probably reflect the long-term course of illness as they can hardly be influenced by the medication we used.

The data on symptom levels are opposed to the findings on functioning: While both groups differed in their psychopathological outcomes, functioning improvements occurred on an equal base. This reflects the outcome of other research that symptom severity and functioning in every-day roles are non-congruent [47]. Only about 10% of patients restored their functioning completely, which is still substantial face to the chronic manifestation of the disorders. About 60% of patients improved in basic dimensions of human existence like communication, living together and self-care. This is encouraging, as we had recruited chronic cases with protracted course of illness.

We know that a comparison between our groups needs to be treated with caution. But it is still necessary to discuss several important implications resulting from the distinction of the two treatment packages and reasons why outpatients had less favorable symptom course but better khat-related outcomes than remitted patients. Our results demonstrate that medication compliance could have been better maintained among outpatients who had received the acute treatment phase and a much more intensive monitoring and psycho-education of medication use during the preventive treatment phase. This is an important selective finding because non-compliance is one of the most important risk factors for relapse. Also khat use among outpatients was kept at a lower level. Only this group of patients and their families received the intensive acute treatment phase, which included psycho-education and assistance to reduce khat use. According to our observations, also the focus on psycho-education for medication during home visits in the preventive phase also led to a more intense psycho-education on khat effects. We also believe that the better adherence to prescribed medication among outpatients can add to the explanation of the less severe khat use in this group. Based on the well-established pharmacological actions of neuroleptic drugs, we can indeed assume that immediate khat effects such as euphoria are dampened under neuroleptic treatment. This might have reduced compulsive use. Based on these results, we hypothesize that medication compliance and khat abstinence can be positively influenced by higher investments in psycho-education, motivational interventions and monitoring.

Over the full project period, low symptom levels could not be maintained with the exception of depressive symptoms. But this symptomatic increase was more pronounced in the outpatient group. This was not expected especially because medication compliance and khat use reductions seemed to be more successful in this group. One potential reason for this symptomatic relapse might be the fact that remitted patients actively maintained contact to service providers (mostly hospital) and outpatients did not. But just the simple fact that a person had been inpatient before, might make it more likely that he/she will search for specialist care in case of a relapse compared to patients who had never had any contact with inpatient treatment facilities before. Another reason might be that our recruitment has produced two groups with a-priori differences. Remitted patients and their families might have lived in better economic situation, had a better formal education, had more knowledge on schizophrenia and its treatment, might have a more western concept of the disorder or even a higher family support or sense of responsibility compared to outpatients [17]. It is difficult to detect potential a-priori differences based on our data. On the one hand, we found no difference concerning the indicators of economic situation and education, but we did find more in-patient treatments, less chaining and restraining and less malnourishment among the patients of the remitted group. These differences possibly reflect poorer care, more stress and inhumane practices, a more chronic and severe course of illness, a higher sensitivity to khat effects and a later start of treatment after illness onset in the outpatient group. These potential differences and their significance need to be addressed in future studies. And as relapse has high costs to patients and families [48] and its prevention is the most important goal in the post-acute treatment phase, future research will need to study the question explicitly which relapse prevention treatment model is the more favorable in a given situation.

Our findings also demonstrate the complex effects of khat use on psychotic outcomes. According to our data and observation, khat use in the acute phase of treatment had a high positive association to the severity of florid symptoms of psychosis. This result replicates the findings reported in the literature that khat use can induce psychotic symptoms or cause exacerbation of pre-existing psychotic disorders [29]. According to our observations, we had managed to motivate outpatients in the first six weeks of the study to stop consuming khat almost completely; this is comparable to the in-patient treatment where patients are not given access to khat. Abstinence is likely to have supported initial treatment effects. However, in the post-acute relapse prevention treatment, this association between khat and symptoms unfolded in more complex interactions. Abstinence motivation of patients and carers eroded in the course of the follow-up period and most patients had returned to khat chewing in the course of the 10 months. But khat use correlated at this point of time inversely with positive psychotic symptoms, i.e. when patients felt well and functioned satisfactory they chewed more khat. These at first sight contradictory correlations may be explained, by the fact that the amount of khat use by a single patient varies greatly over time in a very dynamic system. Factors in this system that increase khat use are addiction and the fact that khat is a common and integral part of social life in the Somali culture. These two factors probably explain why patients restart khat use after remission, i.e. when patients are less psychotic they chew more khat. Factors that decrease khat chewing are external restrictions of access to khat through measures like hog-tying and the desire of the patient to stop khat use. Indeed, we found anecdotal evidence that khat use after remission is noxious and might constitute a risk factor for relapse, because families re-chained patients to restrict khat use when they observed that their psychosis symptoms worsened. This is supported by other studies [32]. Thus, from an ethical perspective, a clinician needs to assume that continuous khat use is a risk factor for a psychotic relapse and has to recommend that khat use is discontinued because even small amounts might be noxious. In this context khat needs to be seen as part of the repeated cycle of relapse and remission, chaining and unchaining of patients. This also demonstrates that the interpretation of an isolated correlation between khat use and psychosis based on data from one single point in time is not meaningful to describe the underlying complex relationship. Antipsychotic medication may help patients to maintain social and occupational functioning and may assist to tolerate consumption within an acceptable range, e.g. by taking the medication in the evening to promote sleep at night. The influence on khat on repeated relapses needs to be explicitly better understood in order to design targeted interventions. Our results also show that short-term intervention strategies to address khat abuse are of limited effect. Our data show that khat addiction is very frequent among psychotic patients and that community-based health services will probably only be effective when its therapy is integral part of the treatment package. Therefore, culturally adequate interventions to address khat addiction urgently need to be developed.

But among users without pre-existing mental disorder khat consumption might actually range from severe abuse with potentially disastrous health effects to more controlled and moderate use patterns that are not or less harmful and that go hand in hand with social integration. The current study was not designed to empirically determine the dynamic boundary between noxious and harmless khat use; future studies are needed to clarify this important question. The difference between noxious and non-noxious khat use probably depends on the amount and hours of use per day, maintaining night sleep, regular food intake *etc.*, all points that need to be explicitly addressed in future studies. According to our anecdotal observations, harmful consequences of khat use among healthy individuals often occur when consuming for several weeks beyond normal amounts (one to two bundles) per day, chewing continuously for more than the culturally sanctioned period of 3 to 5 afternoon hours a day. The consequent inversion of sleeping patterns can be a warning sign of noxious effects on health.

In our work with psychotic patients and their carers in Somalia we observed the common experience of a very severe stigmatization due to mental illness. Patients and carers were excluded and stigmatized in their families and communities and were often abandoned, isolated and impoverished. We, thus, tried to make them feel accepted as valuable members of their communities. The psycho-social assistance turned out to be a vital component of the project intervention. Carers carry a heavy emotional, social and financial burden caring for their mentally ill. Often they reported that the home visits and conversations with project staff were helpful because they found someone to talk to about their stress and thus felt relief. These conversations could then be extended to inform patients and their carers so that they accepted medical recommendations concerning treatment and especially the advice to reduce khat intake.

Our pilot study has a number of limitations. First, our design did not include a control group who received treatment as usual which would have enabled to evaluate the effect of our intervention. Furthermore, both groups of patients entered the study at different points in time; it would have been a great opportunity to recruit one group at hospital intake instead of discharge; this would have made it possible to know whether groups of patients were different before inclusion into the project. In the current project, we probably have compared groups of patients with different a-priori characteristics and courses of symptoms. In future studies, it would be also desirable to include groups of patients without khat abuse and to dismantle effects of different components of the delivered treatment package (i.e. one group without psycho-social interventions). Second, our treatment delivery was also determined by attempts to test feasibility and sustainability rather than current standards of treatment, e.g. using Chlorpromazine and Haloperidol instead of second-generation antipsychotics and using low-dose treatment in all cases - also for those who would have been profited more from higher dosages. However, we followed the WHO guidelines that had been applied in different settings at that time and that were recently published [42]. Third, our treatment packages did not contain important components that are standard in High-Income Countries such as occupational therapy or self-help groups. Self-help groups in LMICs can assist patients and carers to improve social and economic hardships and reduce exclusion and stigmatization in the community [49]. Another challenge that was not addressed in our pilot project is the reintegration of patients into productive life. This might be especially problematic for poorly trained and economically disadvantaged families who constituted the large majority in the present project. Thus, occupational therapy, vocational training and the build-up of income-generating activities on the family level might have helped with the patient’s symptomatic and functional recovery, reduce stigmatization and improve the family’s economic situation. Finally, based on our design and assessment in this sample of patients with chronic psychotic disorder we were not able to determine the exact diagnosis of patients, i.e. whether it was schizophrenia, other long-term psychotic disorders or long-term substance-induced psychotic disorder. In stead, we have focused on selected DSM IV criteria for schizophrenia (characteristic symptoms, social/occupational dysfunctions, duration) to ensure the existence of a long-term psychotic disorder. We believe that the exact diagnosis is not necessary in the context of this study.

Despite limitations this study demonstrates components of effective family-based and outpatient treatment for patients with chronic psychotic disorders in Somalia, where khat abuse among patients is rather the rule than the exception. From a pragmatic point of view, future projects should consider the combination of home visits with psycho-social support and delivery of medication as well as establishing and maintaining contact of the families to the existing in-patient services for referral in case of severe relapses. An inclusion of a self-help as well as an income-generating component for the patients and their families need to be considered because the families live in extreme poverty without much hope to improve their situation and be able to sustain a necessary treatment by own means. Such components might be included during the post-acute treatment phase and should be directed towards patients and their carers. Also patients’ khat addiction must be addressed and consumption be regulated to prevent relapses. Currently there are no evaluated treatment concepts for khat addiction.

## Conclusions

Based on this pilot study, we conclude that despite challenges related to limited local capacities, a reliance on first-generation antipsychotics, despite the missing continuous supervision by trained psychiatrists and despite co-morbid khat abuse, a community-based outpatient mental health treatment is feasible and well accepted under the resource-poor conditions of Somalia. And it shows effective pre to post changes in real-world outcome. Facing the dearth of mental health services and the abundance of inhumane treatment of patients with long-term mental disorders, we conclude that, in spite of the lack of a control group and other methodological shortcomings in this study, community-based treatment is ethically imperative. The key to success of community-based psychiatric intervention seems the trusting relationship of project staff to patients and their carers. This can be established through regular home visits, which provide psycho-social support to patients and carers, which allowed for repeated monitoring and education on psychosis and its treatment as well as the dangers of khat abuse. Due to the reliance on locally available drugs and resources, the costs of the initiated treatments are low in the post-acute phase of treatment. But the extreme poverty prevents that even very cheap treatments be sustained by a large part of the involved families. This problem needs to be addressed by additional income-generating components.

## Competing interests

The authors declare that they have no competing interests.

## Authors’ contributions

MO contributed to the conception and design of the study, the acquisition of data, performed the analysis and contributed to the interpretation of the data, drafted the manuscript, revised the manuscript and approved the final manuscript. BL contributed to the conception and design of the study, the acquisition of data and approved the final manuscript. WP contributed to the conception and design of the study, the acquisition of data and approved the final manuscript. FAH contributed to the acquisition of data and approved the final manuscript. AMW contributed to the acquisition of data and approved the final manuscript. AO contributed to the acquisition of data and approved the final manuscript. JS contributed to the acquisition of data and approved the final manuscript. AM contributed to the acquisition of data and approved the final manuscript. TE contributed to the conception and design of the study, the analysis and interpretation of data, revised the manuscript and approved the final manuscript. All authors read and approved the final manuscript.

## Supplementary Material

Additional file 1 **Table S1.** Medication side effects at follow-up. The table reports means and standard deviations as well as percentages and N. Results of group comparisons between outpatients and remitted patients are reported in the last column. ^1^ missing data: 16 outpatients, 13 remitted patients. ^2^ missing data: 17 outpatients, 12 remitted patients. Table S2: Khat use indicators during the project. We report percentages and N or means and standard deviations before enrollment and at follow-up. ^1^ missing data: 15 outpatients, 17 remitted patients (10 discharged, 7 community). ^2^ missing data: 13 outpatients, 16 remitted patients (9 discharged, 7 community). ^3^ missing data: 16 outpatients, 16 remitted patients (9 discharged, 7 community). ^4^ missing data: 17 outpatients, 16 remitted patients (10 discharged, 6 community). ^5^ missing data: 16 outpatients, 16 remitted patients (10 discharged, 6 community). Click here for file
