# Supplementary material for: A pilot study on community-based outpatient treatment for patients with chronic psychotic disorders in Somalia: Change in symptoms, functioning and co-morbid khat use
Source: Int J Ment Health Syst. 2012 Jul 2;6:8. doi: 10.1186/1752-4458-6-8 (PMC3527287; doi:10.1186/1752-4458-6-8)
Supplement: Additional file 1 — Table S1. Medication side effects at follow-up. The table reports means and standard deviations as well as percentages and N. Results of group comparisons between outpatients and remitted patients are reported in the last column. 1 missing data: 16 outpatients, 13 remitted patients. 2 missing data: 17 outpatients, 12 remitted patients. Table S2: Khat use indicators during the project. We report percentages and N or means and standard deviations before enrollment and at follow-up. 1 missing data: 15 outpatients, 17 remitted patients (10 discharged, 7 community). 2 missing data: 13 outpatients, 16 remitted patients (9 discharged, 7 community). 3 missing data: 16 outpatients, 16 remitted patients (9 discharged, 7 community). 4 missing data: 17 outpatients, 16 remitted patients (10 discharged, 6 community). 5 missing data: 16 outpatients, 16 remitted patients (10 discharged, 6 community). [file 1752-4458-6-8-S1.docx]

Table S1: Medication side effects at follow-up. The table reports means and standard deviations as well as percentages and N. Results of group comparisons between outpatients and remitted patients are reported in the last column.

|  |  | All patients  (30) | Outpatients  (17) | Remitted Patients  (13) | p |
| --- | --- | --- | --- | --- | --- |
| Any Side Effect^1^ | Frequency of any side effect | 86.7%  (26) | 82.4%  (14) | 92.3%  (12) | .613 |
|  | Average number of side effects | 3.11  (2.31) | 2.65  (2.09) | 3.77  (2.52) | .193 |
|  | Severity | 1.80  (0.63) | 1.83  (0.63) | 1.77  (0.65) | .794 |
| Dyskinesia | Frequency | 43,3%  (13) | 35.3%  (6) | 53,8%  (7) | .310 |
|  | Severity | 2.00  (0.82) | 1.67  (0.52) | 2.29  (0.95) | .234 |
| Akatisia | Frequency | 23.3%  (7) | 17.6%  (3) | 30.8%  (4) | .666 |
|  | Severity | 2.00  (0.89) | 2.00  (0) | 2.00  (1.16) | 1.000 |
| Dry mouth | Frequency | 43.3%  (13) | 35.3%  (6) | 53.8%  (7) | .310 |
|  | Severity | 1.33  (0.65) | 1.20  (0.45) | 1.43  (0.79) | .755 |
| Blurred vision^1^ | Frequency | 20.7%  (6) | 6.3%  (1) | 38.5%  (5) | .064 |
|  | Severity | 1.83  (0.98) | 1  (0) | 2  (1.00) | .667 |
| Increased appetite with weight gain | Frequency | 33.3%  (10) | 23.5%  (4) | 46.2%  (6) | .255 |
|  | Severity | 1.67  (0.71) | 1.67  (1.16) | 1.67  (0.52) | .905 |
| Constipation | Frequency | 26.7%  (8) | 35.3%  (6) | 15.4%  (2) | .407 |
|  | Severity | 2.14  (0.69) | 2.40  (0.55) | 1.50  (0.71) | .190 |
| Problems with urine control | Frequency | 13.3%  (4) | 11,8%  (2) | 15.4%  (2) | 1.000 |
|  | Severity | 2.00  (1.16) | 2.00  (1.41) | 2.00  (1.41) | 1.000 |
| Allergic skin rash | Frequency | 6.7%  (2) | 0%  (0) | 15.4%  (2) | .179 |
|  | Severity | 1.00  (0) | - | 1.00  (0) | - |
| Photosensitivity | Frequency | 6.7%  (2) | 0%  (0) | 15.4%  (2) | .179 |
|  | Severity | 1.00  (0) | - | 1.00  (0) | - |
| Sedation^2^ | Frequency | 55.2%  (16) | 47.1%  (8) | 66.7%  (8) | .296 |
|  | Severity | 1.93  (0.83) | 2.00  (0.89) | 1.88  (0.84) | .852 |
| Increased saliva flow^2^ | Frequency | 20.7%  (6) | 23.5%  (4) | 16.7%  (2) | 1.000 |
|  | Severity | 1.00  (0) | 1.00  (0) | 1.00  (0) | 1.000 |
| Fever/Hyperthermia^2^ | Frequency | 3.4%  (1) | 5.9%  (1) | 0 | 1.000 |
|  | Severity | 3.00  (0) | 3.00  (0) | - | - |
| Jaundice^2^ | Frequency | 3.4%  (1) | 5.9%  (1) | 0 | 1.000 |
|  | Severity | 2.00  (0) | 2.00  (0) | - | - |
| Other side effects^2^ | Frequency | 17.2%  (5) | 17.6%  (3) | 16.7%  (2) | 1.000 |
|  | Severity | 1.75  (0.96) | 2.50  (0.71) | 1.00  (0) | .333 |

^1^ missing data: 16 outpatients, 13 remitted patients

^2^ missing data: 17 outpatients, 12 remitted patients

Table S2: Khat use indicators during the project. We report percentages and N or means and standard deviations before enrollment and at follow-up.

|  |  | Outpatients (17) | Remitted Patients (18) | p |
| --- | --- | --- | --- | --- |
| Before enrollment | Current khat use (last month)^1^ | 76.5%  (13) | 58.8%  (10) | .271 |
|  | Days of khat use in the week before interview^2^ | 3.8  (2.8) | 1.4  (2.1) | .013 |
|  | Bundles of khat per week  (last month)^3^ | 4.6  (6.1) | 1.0  (1.6) | .128 |
| Follow-up | Current khat use (last month)^4^ | 58.8%  (10) | 93.8%  (15) | .039 |
|  | Days of khat use in the week before the interview^5^ | 2.8  (3.2) | 3.6  (3.0) | .460 |
|  | Bundles of khat per week (last month)^4^ | 3.1  (6.1) | 4.1  (3.9) | .594 |

^1^ missing data: 15 outpatients, 17 remitted patients (10 discharged, 7 community)

^2^ missing data: 13 outpatients, 16 remitted patients (9 discharged, 7 community)

^3^ missing data: 16 outpatients, 16 remitted patients (9 discharged, 7 community)

^4^ missing data: 17 outpatients, 16 remitted patients (10 discharged, 6 community)

^5^ missing data: 16 outpatients, 16 remitted patients (10 discharged, 6 community)
